# The effect of feeding order of forage and oats on metabolic and digestive responses related to gastric emptying in horses

**DOI:** 10.1093/jas/skae368

**Published:** 2024-12-04

**Authors:** Rasmus Bovbjerg Jensen, Ingrid Hornnes Walslag, Caroline Marcussen, Nana Wentzel Thorringer, Peter Junghans, Nicole Frost Nyquist

**Affiliations:** Department of Animal and Aquacultural Sciences, Norwegian University of Life Sciences, Ås, Norway; Department of Paraclinical Sciences, Norwegian University of Life Sciences, Ås, Norway; Department of Veterinary Clinical Sciences, University of Copenhagen, Frederiksberg, Denmark; Department of Animal and Aquacultural Sciences, Norwegian University of Life Sciences, Ås, Norway; Institute of Nutritional Physiology ‘Oskar Kellner’, Research Institute for Farm Animal Biology (FBN), Dummerstorf, Germany; Department of Paraclinical Sciences, Norwegian University of Life Sciences, Ås, Norway

**Keywords:** ^13^C acetic acid, cecal pH, equine, glucose, insulin, stable isotopes

## Abstract

Feeding order of forage and concentrate might affect gastric emptying and subsequently digestion in horses. The objective of this study was to measure gastric emptying in combination with metabolic and digestive responses in the plasma and cecum, respectively, when changing the feeding order of oats (**O**) and hay (**H**) (oats first, then hay: O–H vs. hay first, then oats: H–O). Four cecum cannulated horses were used in a 2 × 2 crossover design, with two 12-d periods consisting of 10 d of diet adaptation and 2 d of data collection. Hay was fed at 0600, 1400, and 2000 hours, while oats were fed in the morning either 15 min before or 1 h after feeding hay. On days of data collection, baseline samples were collected before feeding 1.4 kg dry matter (**DM**) hay and 474 g DM oats (0.4 g starch/kg body weight), and data were collected until 8 h after feeding. Gastric emptying of oats was estimated using the ^13^C acetic acid breath test, where breath samples were analyzed for a ^12^C:^13^C ratio after administration of ^13^C acetic acid mixed with oats. Gastric emptying coefficient (**GEC**), time where half of the total cumulative recovery of ^13^C was excreted (***t***_**1/2**_), and time where the maximal amount of ^13^C was excreted (***T***_**max**_) were calculated. Samples of blood and cecal fluid were collected at hourly intervals. Blood plasma was analyzed for glucose and insulin, and baseline concentrations, peak concentrations, time of peaks, and area under the curves were calculated. A pH probe was placed in the cecum measuring pH every minute to find minimum pH and time to reach minimum pH. Hourly cecal samples were analyzed for pH and short-chained fatty acid (**SCFA**) concentrations. Results from the ^13^C acetic acid breath test indicated that feeding order affected gastric emptying, as *T*_max_ was longer (*P* = 0.004) when feeding H–O (2.18 h) than O–H (1.09 h), but there was no effect on the GEC and *T*_1/2_. No effect of feeding order was found for plasma glucose and insulin measures. Feeding order had no effect on minimum pH, but the time to reach minimum pH increased (*P* = 0.014) from 170 min for O–H to 280 min for H–O, and average pH was lower in the intervals 0–170 min (*P* = 0.006) and 170–280 min (*P* = 0.006) for O–H than H–O. In general, the time of sampling had a larger effect on SCFA concentrations than feeding order. In conclusion, this study indicates that feeding order affected gastric emptying, and the digestive and metabolic responses were more clearly reflected in cecum pH than in plasma glucose and insulin.

## Teaser

Basic knowledge of how diet composition and feeding management affects normal gastrointestinal function and digestion in healthy horses is needed to avoid feed-related diseases. This study indicates that the feeding order of forage and concentrate affects the digestive and metabolic responses in the cecum and plasma of horses.

## Introduction

Horses have evolved as grazing nonruminant herbivores with a relatively small stomach and a highly specialized hindgut (cecum and colon) capable of fermenting fibrous feeds (Janis, 1976). However, domestication has altered the feeds consumed by horses, and today conserved forages and nonfibrous starch-rich concentrates make up the ration as a substitute or supplement to grazing (Julliand et al., 2006; Harris et al., 2017). It is recommended to not feed more than 1 g starch/kg body weight (**BW**) per meal (Coenen et al., 2011) as excessive amounts of starch might have negative health effects on horses, thereunder dietary-induced laminitis (Garner et al., 1975), colic (Hudson et al., 2001), gastric ulcers (Luthersson et al., 2009), and increased behavioral reactivity (Bulmer et al., 2019). Knowledge of how diet composition and feeding management affect normal gastrointestinal function and digestion in healthy horses is needed to understand how domesticated horses should be fed to avoid the development of feed-related diseases.

Previous studies indicate that factors such as diet composition (Wyse et al., 2001) and meal size (Métayer et al., 2004) might affect gastric emptying in horses. When using the ^13^C-octanioic acid breath test to quantify gastric emptying, Wyse et al. (2001) found a slower gastric emptying when increasing the amount of oil in the diet of ponies. Métayer et al. (2004) measured gastric emptying with scintigraphy and found that a larger meal (700 g/100 kg BW) increased gastric emptying compared with a smaller meal (300 g/100 kg BW). This indicated that retention of feed in the stomach and passage of feed to the small intestine and subsequently the hindgut of the horse is affected by diet composition and feeding management.

Studies have shown that microbial fermentation of starch occurs in the stomach of horses when feeding concentrate, resulting in the production of short-chained fatty acids (**SCFAs**) and lactate (de Fombelle et al., 2004; Varloud et al., 2004). In theory, a longer retention time of concentrate in the stomach might potentially increase starch fermentation and affect the amount of starch passing to the small intestine. Plasma glucose and insulin increase after feeding a starch rich meal (Vervuert et al., 2003), but the responses might be affected by feeding status (access to hay before and/or after feeding a standardized meal of cracked corn [**CC**]) (Vervuert et al., 2008). However, Vervuert et al. (2008) found limited effects on the glycemic and insulinemic responses when investigating the feeding order of oats and chopped alfalfa. The pre-cecal digestibility of starch varies depending on the source of starch, amount of starch, and processing of the grain (Meyer et al., 1995). The apparent total tract digestibility of starch is, however, close to 100% as starch escaping the stomach and small intestine is fermented in the hindgut (Julliand et al., 2001). Feeding order might also affect when, and what nutrients reach the hindgut. Philippeau et al. (2009) found that feeding ground barley before hay resulted in a lower cecal pH compared with the opposite feeding order. Furthermore, Jensen et al. (2012) found no differences in minimum pH, but minimum pH was reached faster when feeding pelleted barley before hay compared with the opposite feeding order. These results indicate that feeding order might affect gastric emptying and the following digestive responses in the hindgut.

The interactions of diet composition and feeding management on digestion can be challenging to investigate, and gastric emptying, metabolic, and digestive responses have not been investigated simultaneously in horses. Therefore, the objective of this study was to measure gastric emptying in combination with metabolic and digestive responses in the plasma and cecum of horses, respectively, when changing the feeding order of oats and haylage. It was hypothesized that feeding oats before forage would result in 1) a rapid gastric emptying of oats (measured by the ^13^C-octanioic acid breath test), 2) a faster metabolic response in plasma glucose and insulin, and 3) a faster increase in cecal SCFA concentrations and decrease in pH, compared with feeding hay before oats.

## Materials and Methods

### Experimental design

All housing, management, and experimental procedures followed the laws and regulations for experimental animals in Norway (i.e., Regulations on the Use of Animals in Experiments, July 2015), and the experiment was approved by the Norwegian Food Safety Authority (FOTS ID 24451). The experiment was designed as a 2 × 2 crossover design with two experimental periods of 12 d, where feeding order of hay and oats differed in the two periods. Each period consisted of 10 d of diet adaptation and 2 d of data collection. Blood and cecal samples were collected on day 11 and gastric emptying was measured on day 12 in each period.

### Animals, housing, and management

Four cecum-cannulated Norwegian cold-blooded trotter horse geldings, age ranging from 15 to 24 yr of age, were included in the experiment. Their BW was 566 ± 41 kg. All horses are routinely examined by a veterinarian for general health, dental examination, and yearly vaccinations. The cecal cannulas (length ~15 cm; 40 mm outer diameter, and 30 mm inner diameter) made of polyvinyl chloride were permanently fitted at the base of the cecum close to the ileocecal junction more than 10 yr before the experiment. The horses were housed in an insulated barn in individual stalls (3 × 3 m) with rubber mats and wood shavings for bedding. During the adaptation periods, all horses were turned out in a shared gravel paddock after morning feedings from 1000 to 1400 hours and in the afternoon from 1600 to 2000 hours. During days of sampling, the horses were kept inside their stalls.

### Feedstuffs and feeding

All horses were fed the same diets in three meals per day at 0600, 1400, and 2000 hours. Nonprocessed whole oats (*Avena sativa,* Champion, Felleskjøpet Forutvikling, Trondheim, Norway) were fed once per day (474 g dry matter [**DM**]/d; 0.4 g starch/kg BW) in the morning. The haylage, from one batch of late-cut grass consisting mostly of Timothy (*Phleum pratense*), was fed (6,900 g DM/d) divided into three meals of 1.5, 3, and 3 kg as fed. Oats and haylage were fed separately; either haylage first and oats 1 h later (H–O), or oats first followed by haylage 15 min later (O–H), to allow the horses enough time to finish the first meal (oats or haylage) before feeding the next. In each 12 d period, two horses were fed either H–O or O–H, and feeding order was then changed in the next period.

During the adaptation periods, the morning meal included 30 g/d sodium chloride and 100 g/d of a commercial mineral and vitamin supplement (Champion Multitilskudd, Felleskjøpet Forutvikling, Trondheim, Norway) consisting, per kg as fed, of Ca, 100 g; P, 70 g; Mg, 32 g; NaCl, 50 g; Cu, 840 mg; Zn, 2,830 mg; Mn, 1,530 mg; Fe, 2,460 mg; I, 18 mg; Co, 6 mg; Se, 10.2 mg; vitamin A, 107,000 I.U.; vitamin D, 11,300 I.U.; vitamin E, 9,600 mg; vitamin B1, 260 mg; vitamin B2, 120 mg; vitamin B6, 100 mg; vitamin B12, 0.8 mg; niacin, 270 mg; folic acid, 150 mg; biotine, 15 mg; and vitamin C, 270 mg. The horses had access to water ad libitum by means of automatic water troughs in the separate stalls and water troughs in the gravel paddock.

### Sampling procedures

#### Feedstuffs

Samples of haylage and oats were collected from each period and stored for later analysis of chemical composition.

#### Gastric emptying

Gastric emptying was measured on day 12 in each period (Wyse et al., 2001; Sutton et al., 2003; Jensen et al., 2015). One gram of ^13^C-acetate (98 at % ^13^C, Sigma Aldrich, St. Louis, USA) was mixed into the oats with ~20 mL of water to make sure the container with isotope was emptied completely. Before feeding oats and the administration of ^13^C-acetate to the horses, three baseline breath samples (at −10, −7, and −5 min) from each horse were collected. After feeding, breath samples were collected every 30 min for 4 h, then every hour for 4 h, resulting in 15 samples per horse per period. An anesthetic rubber mask (anesthetic mask rubber 50 mm, Jørgen Kruuse A/S, Langeskov, Denmark) connected to a two-way non-rebreathing valve system (Hans Rudolph, Inc., Kansas City, USA) was used for the sampling. The mask was held over one nostril and the other nostril was covered with a hand when the horse exhaled. It took ~30 s to fill up one 1 liter breath bag (Wagner Analysen Technik GmbH, Bremen, Germany) with exhaled air. Collected samples were stored at room temperature until analysis.

#### Blood samples

Blood samples were collected by jugular vein puncture on day 11 in each period, once before feeding oats and then every hour for eight consecutive hours after feeding oats. Immediately after sampling, blood samples were collected in 10 mL heparin tubes (BD Vacutainer, Becton, Dickinson and Company, Franklin Lakes, USA) and centrifuged at 3,000 x g for 10 min (Heraeus labofuge 300, Thermo Fisher Scientific, Waltham, USA). After centrifuging, plasma was harvested and stored at −20 °C for later analysis of plasma glucose and insulin concentrations.

#### Cecal samples and pH measurements

Cecal samples were collected at the same time as the blood samples on day 11, and ~100 mL of cecal fluid was collected with a 400 mL syringe through the cecal cannula. Cecal fluid pH was measured with a pH electrode (Sentix 41, WTW, Weilheim, Germany) immediately after sampling. A subsample of 9.5 mL was mixed with 0.5 mL of formic acid for preservation and stored at 3 °C for later analysis of SCFA concentrations. Additionally, a pH electrode (Sentix 41, WTW) attached to the data logger (ProfiLine 340i, WTW) was placed directly in the cecum through the permanent cecal cannula measuring and logging pH continuously throughout the sampling day. Measurements of cecal pH were saved on the pH logger once every minute for 9 h.

## Analysis

Haylage and oats from each period were ground to 1 mm (mixer mill MM 301, Retsch GmbH, Haan, Germany) before analysis. For starch, oats were milled to pass a 0.5-mm screen before analysis. DM content was determined by drying to a constant weight (24 h at 103 °C), and samples were incinerated at 550 °C for ~16 h for crude ash determination. Neutral and acid detergent fibers were assayed with a heat-stable amylase and expressed without residual ash (aNDFom and aADFom) by the filter bag technique described by ANKOM (ANKOM 2017a, b). Nitrogen was determined by the Kjeldahl technique using a Kjeltec TM 8400 (FOSS, Hillerød, Demark) and crude protein was calculated as 6.25 × N. Water-soluble carbohydrates (**WSC**) were determined as described by Randby et al. (2010). Starch was measured according to the Association of Official Analytical Chemists (**AOAC**, method 996.11.) by using heat-stable α-amylase. The concentrations of SCFA were determined by gas chromatography (Trace 1300 GC, Thermo Fisher Scientific).

The breath contained in bags was analyzed for ^13^C enrichment of CO_2_ by means of the infrared isotope analyzer (IRIS, Wagner Analysen Technik GmbH). Plasma glucose was analyzed by the hexokinase method (Tietz, 1995), and insulin was analyzed using the ELISA test (Mercodia AB, Uppsala, Sweden).

### Calculations and statistical analysis

#### Gastric emptying

The ^13^C kinetics and further calculations were evaluated based on the ratio of ^13^C:^12^C in breath CO_2_ (Ghoos et al., 1993; Braden et al., 1995). The ratio was expressed as the relative δ^13^C (delta) value (in ‰). For further calculations, the δ^13^C values were converted to ^13^C abundances AP (in atom %):


$$\begin{array}{l} AP=\frac{R\times \left(\frac{\delta {}_{}^{13}C}{1000+1}\right)}{1+R\times \left(\frac{\delta {}_{}^{13}C}{1000+1}\right)}\times 100 \end{array}$$ (1)

where R is the international Pee Dee Belemnite standard (R = 0.0112372, Craig, 1957).

For practical purposes, the ^13^C enrichments **APE** (in atom %) were used:


$$\begin{array}{l} APE=AP_{Sample}-AP_{Baseline} \end{array}$$ (2)

where AP_Baseline_ is the ^13^C abundance of excreted breath CO_2_ before ^13^C-acetate administration in the fasting state and AP_Sample_ is the ^13^C abundance at the time after administration of the ^13^C-acetate and during feeding.

The mean daily CO_2_ production of the horses is estimated as follows (Jensen et al., 2015):


$$\begin{array}{l} CO_{2}\;production=4.8\;\left(\frac{liters}{day}\;per\;kg\;BW\right) \end{array}$$ (3)

The CO_2_ production is converted from liter/day to mol per hour:


$$\begin{array}{l} \;\;\;\;\;CO_{2}\;production\;\left(mol/h\right)=\frac{CO_{2}\;\;production\;\left(liters/day\right)}{22.4\;\left(liters/mol\right)\times 24\;\left(h/day\right)} \end{array}$$ (4)

where 22.4 liters/mol is the molar volume of gases.

The amount of ^13^C excreted in breath CO_2_ within a time interval Δt is given by


$$\begin{array}{l} \;\;\;{}^{13}C\;amount\;\left(mmol\right)=\;\frac{APE\left(atom\;\%\right)\;\times \;CO_{2}\;production\;\;\left(mmol\right)\;\;\;}{100}\times 1000 \end{array}$$ (5)

where Δt is the time interval (h) between two consecutive breath samples collected for ^13^C measurements. APE is the ^13^C enrichment of breath CO_2_ of each sample. The cumulative ^13^C excretion (mmol) is calculated by successive adding of the previous ^13^C amount (mmol) and the current one. Thus, the cumulative ^13^C excretion in breath CO_2_ (% of the ^13^C dose) can be expressed by:


$$\begin{array}{l} cumulative\;{}_{}^{13}C\;\;excretion\;\left(\%\;of\;{}_{}^{13}C\;dose\right)=\frac{cumulative\;{}_{}^{13}C\;excretion\;\left(mmol\right)}{{}_{}^{13}C\;dose\;\left(mmol\right)\;\;\;}\times 100 \end{array}$$ (6)

where the ^13^C dose (in mmol) administered to the horses was calculated by the following equation:


$$\begin{array}{l} \;\;\;\;\;\;\;\;\;\;\;\;\;\;\;\;\;\;\;\;\;\;\;\;\;\;\;\;\;\;\;\;\;\;\;\;\;\;\;\;\;{}_{}^{13}C\;dose\;\left(mmol\right)=\frac{{}_{}^{13}C\;dose\;of\;{}_{}^{13}C\;\;acetate\;\left(g\right)}{83\;\left(g/mol\right)}*1000 \end{array}$$ (7)

with the molar mass of ^13^C-acetate of 83 g mol^−1^.

Using equations (5) and (7) it is possible to calculate the percentage of ^13^C excretion in breath CO_2_ per hour percentage of 13C excretion in breath CO2 per hour (**PDR**), i.e., the ^13^C excretion (mmol per h) referred to the administered ^13^C dose (in mmol).


$$\begin{array}{l} \;\;\;\;\;\;\;\;\;\;\;\;\;\;\;\;\;\;\;\;\;\;\;\;\;\;\;\;\;\;\;\;\;\;\;\;\;\;\;\;\;\;\;\;\;\;\;\;\;\;\;\;\;\;\;\;\;\;\;\;\;PDR=\frac{{}_{}^{13}C\;excretion\;{\left(mmol/h\right)}_{}}{{}_{}^{13}C\;dose\;\left(mmol\right)}*100 \end{array}$$ (8)

Thereafter, PDR was plotted against the time. The PDR-t curve may be fitted using the formula:


$$\begin{array}{l} \;\;\;\;\;\;\;\;\;\;\;\;\;\;\;\;\;\;\;\;\;\;\;\;\;\;\;\;\;\;\;\;\;\;\;\;\;\;\;\;\;\;\;\;\;\;\;\;\;\;\;\;\;\;\;\;\;\;\;\;\;\;\;\;\;\;\;\;\;\;\;\;\;\;\;\;\;\;\;\;\;\;\;\;\;\;\;\;\;\;\;\;\;\;\;\;\;y=at^{b}e^{-ct} \end{array}$$ (9)

where y is PDR (% per h), t is the time (h) and a, b, and c are regression constants. The data was fitted to the model equation (9) using the NLIN procedure in SAS (9.4) and values for a, b, and c were obtained.

The following quantities may be calculated:


$$\begin{array}{l} GEC=ln\left(a\right) \end{array}$$ (10)

$$\begin{array}{l} t_{1/2}=Gamma.inv(0.5;b+1;1/c \end{array}$$ (11)

The formula for *t*_1/2_ is calculated in Excel.


$$\begin{array}{l} \;\;\;\;\;\;\;\;\;T_{max}=b/c \end{array}$$ (12)

$$\begin{array}{l} \;\;\;\;\;RF=\frac{cumulative\;13C\;excretion\;\left(mmol\right)}{\;\;\;13C\;dose\;\left(mmol\right)}*100 \end{array}$$ (13)

where the gastric emptying coefficient (**GEC**) is an overall index of gastric emptying, *t*_1/2_ is the time, where half of the total cumulative recovery of ^13^C is excreted, *T*_max_ is the time where the maximal amount of ^13^C is excreted, and **RF** is the cumulative ^13^C excretion (in % of the ^13^C dose) at the plateau value, which is approached at 8 h.

#### Blood samples

Baseline concentration, peak concentration, time of peak, and area under the curves (**AUC**) above baseline (without considering area beneath) were performed for plasma glucose and insulin in GraphPad Prism (version 8.1.2, GraphPad Software, San Diego, USA).

#### Cecal samples and pH measurements

First, the continuously measured cecal pH was averaged into 10-min intervals. Thereafter, minimum cecal pH and time to reach minimum pH were found. Based on the time for reaching minimum pH, three time intervals were decided and average pH within the intervals 0–170, 180–280, and 290–540 min were calculated. SCFA were measured hourly and molar proportions (mol/100 mol) of individual SCFA were calculated.

#### Statistical analysis

All data was confirmed to be within normal distribution before all statistical analyses were performed using the proc mixed procedure in SAS (Version 9.4, SAS Institute Inc., Cary, USA). The model compromised the fixed effect of feeding order (H–O or O–H) and the random effect of horse, when analyzing the responses from gastric emptying measurements, metabolic responses in plasma, and responses derived from the continuously measured pH. Measurements from hourly cecal samples were analyzed as repeated measurements, where the model comprised the fixed effect of feeding order (O–H or H–O) and time (time after feeding: 0–9 h) and interactions (feeding order and time) and the random effect of horse. Serial correlation over horse was modeled using a spatial Gaussian correlation structure. Results are presented as least square means with standard error of the mean (**SEM**) as a measure of variance, and effects were considered significant if *P* < 0.05.

## Results

### Feedstuffs and feeding

DM and chemical composition of haylage and oats used in the experiment are shown in Table 1. Exact eating times were not recorded, but all horses consumed the feeds within the allocated time and adding the dose of ^13^C-actetate appeared to not influence feed intake (the oats were consumed right after feeding).

**Table 1.** Dry matter (DM, g/kg) and chemical composition (g/kg DM) of haylage and oats used in the experiment

	Hay (*N* = 2)	Oats (*N* = 2)
Dry matter	925	947
Ash	56.7	25.1
aNDFom^1^	635	319
aADFom^1^	373	149
ADL	32.4	24.6
Crude protein	85	126
Starch	-	442
Water-soluble carbohydrates	103	44

Abbreviation: ADL = acid detergent lignin.

^1^Neutral and acid detergent fiber assayed with heat-stable amylase and expressed without residual ash.

### Gastric emptying

**Figure 1.**
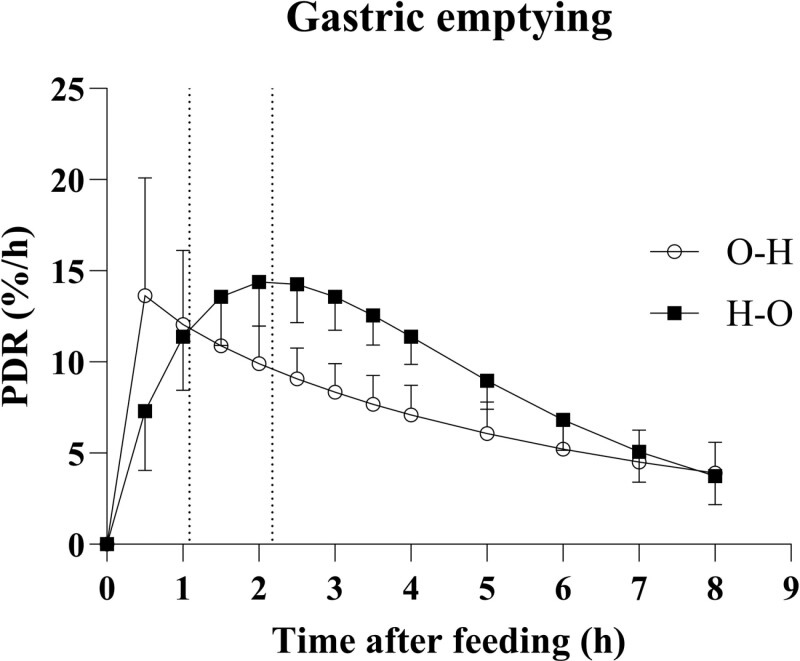
Percentage dose recovered per hour (PDR/h) of breath ^13^C over 8 h measurements following feeding oats mixed with a dose of ^13^C-acetate before (○ Oats-Hay) or after (■ Hay-Oats) feeding hay. Vertical dotted lines represent the time to peak breath ^13^C-excretion (*T*_Max_, *P* = 0.004). Values are presented as least square means ± standard error of the mean.

The percentage of the ^13^C dose recovered in breath per hour (PDR, %/hour) against time is shown in Figure 1. There was no effect of feeding order on GEC, *t*_1/2,_ and RF, but *T*_Max_ was lower (*P* = 0.0044) when feeding O–H than H–O (Table 2).

**Table 2.** Gastric emptying coefficient (GEC), time to peak breath ^13^C-excretion (*T*_Max_), time where half of the marker ^13^C is exhaled (*t*_1/2_) and recovery factor (RF) of ^13^C when feeding either O before (O–H) or after (H–O) H. Values are presented as least square means ± standard error of the mean

	O–H	H–O	SEM	*P*-value
GEC	2.61	2.88	0.18	0.36
*T*_max_	1.09	2.18	0.19	0.004
*t*_½_	4.92	3.73	0.91	0.42
RF	58.7	73.3	5.6	0.16

Values within a row are different if *P* < 0.05.

Abbeviation: SEM = standard error of the mean.

### Blood samples

**Figure 2.**
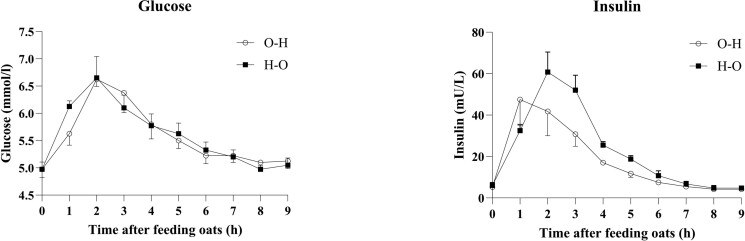
Plasma glucose and insulin curves when either oats before (O–H) or after (H–O) hay. Baseline, peak, time to peak, and AUC for plasma glucose and insulin were not affected by feeding order (*P* > 0.05). Values are presented as least square means ± standard error of the mean.

Plasma glucose and insulin increased after feeding and then returned to baseline levels at the end of measurements (Figure 2). There was no effect of feeding order on baseline concentrations, peak concentrations, time at peak concentrations, or AUC for glucose or insulin (Table 3).

**Table 3.** Baseline concentration, peak concentration, time of peak (hours after feeding oats), and area under the curve (AUC) of glucose (mM/liter) and insulin (mU/liter) when feeding either oats before (O–H) or after (H–O) hay. Values are presented as least square means ± standard error of the mean

	O–H	H–O	SEM	*P*-value
Glucose				
Baseline	4.98	4.98	0.14	1.00
Peak	6.75	6.73	0.28	0.94
Time peak (h)	2.25	2.00	0.34	0.39
AUC	5.94	6.23	0.96	0.79
Insulin				
Baseline	5.25	6.00	1.17	0.68
Peak	55.0	63.0	9.45	0.54
Time peak (h)	2.00	2.50	0.35	0.18
AUC	127.9	168.6	20.9	0.24

Values within a row are different if *P* < 0.05.

Abbreviation: SEM = standard error of the mean.

### Cecal samples and pH measurements

**Figure 3.**
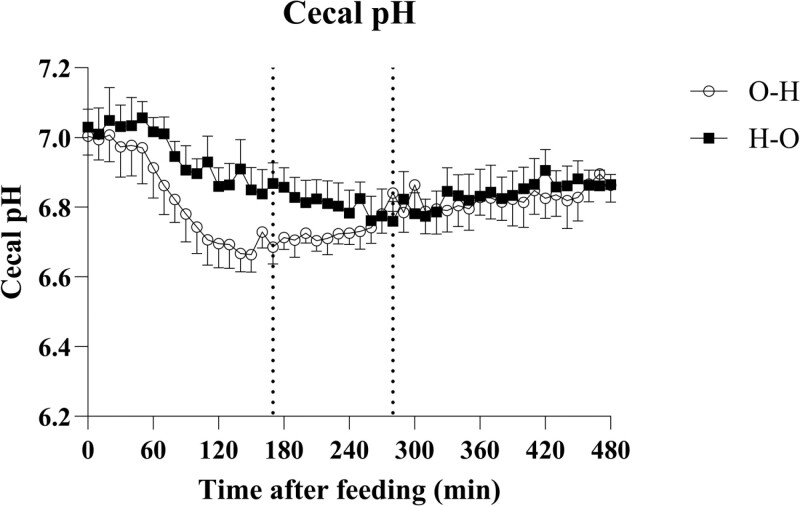
Cecal pH was measured every 10 min through 480 min (9 h) when feeding either O–H or H–O. Minimum pH was not affected by feeding order (*P* > 0.005), but the time for reaching minimum pH was (vertical dotted lines represent O–H: 170 min vs. H–O: 280 min, *P* = 0.014). Values are presented as least sqaure means ± standard error of the mean.

Cecal pH decreased after feeding and increased again after reaching a minimum (Figure 3). There was no effect of feeding order on minimum pH, but minimum pH was reached faster (*P* = 0.014) when feeding O–H than H–O (Table 4). Cecal pH was lower in the time intervals 0–170 min (*P* = 0.006) and 180–280 min (*P* = 0.005) when feeding O–H than H–O (Table 4).

**Table 4.** Minimum pH, time for minimum pH (min after feeding oats), and average pH in the interval 0–170,180–280, and 290–540 min when feeding either O–H or H–O. Values are presented as least square means ± standard error of the mean

	O–H	H–O	SEM	*P*-value
Minimum pH	6.64	6.72	0.059	0.14
Time of minimum pH	170	280	19.8	0.014
0–170	6.83	6.95	0.058	0.006
180–280	6.79	6.89	0.052	0.005
290–540	6.84	6.85	0.052	0.89

Values within a row are different if *P* < 0.05.

Abbreviation: SEM = Standard error of the mean.

**Figure 4.**
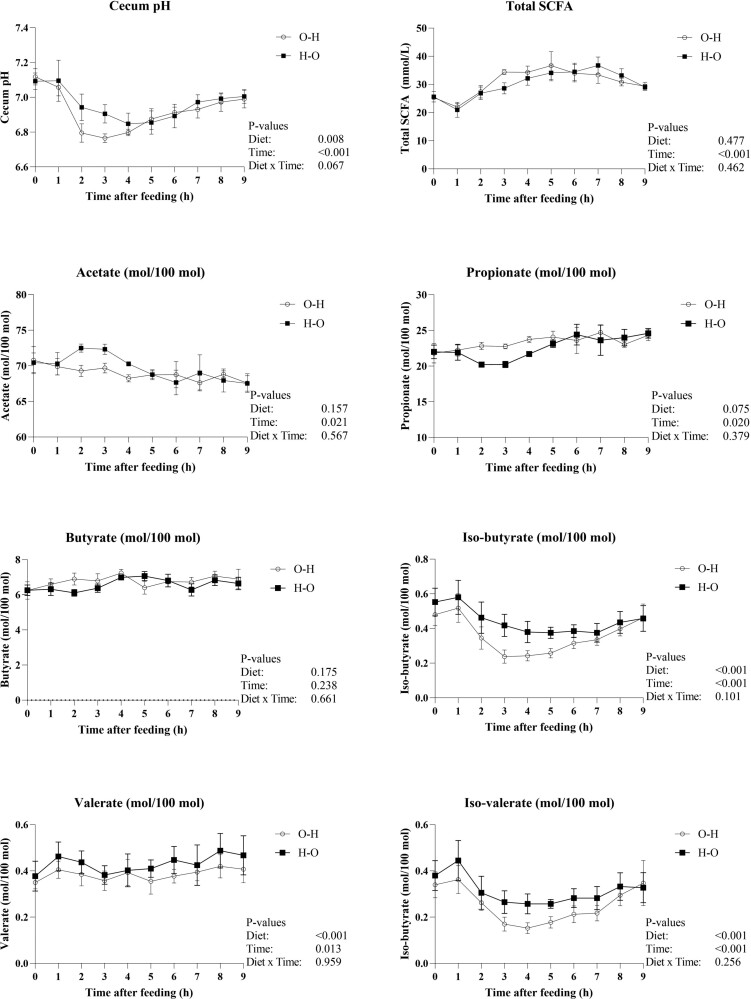
Average of cecum pH, total SCFA, and molar proportions (mmol/100 mmol) of individual SCFA in samples of cecal fluid for all four horses in both feeding periods. One sample per hour per horse, a total of nine samples per horse per period. One curve for the feeding order O–H, and one curve for feeding H–O.

Cecal pH and SCFA concentrations measured in the hourly samples are shown in Figure 4. Cecal pH decreased (*P* < 0.001) after feeding and increased again after reaching a minimum, and cecal pH was lower (*P* = 0.008) when feeding O–H than H–O. The concentration of total SCFA increased (*P* < 0.001) after feeding, but there was no effect on the feeding order. The proportion of acetate decreased (*P* = 0.021) at the expense of propionate which increased (*P* = 0.020) over time. Butyrate was not affected by feeding order or time. Isobutyrate and isovalerate were higher (*P* < 0.001) when feeding H–O than O–H, and the concentrations decreased (*P* < 0.001) after feeding and increased again after reaching a minimum. Valerate was higher (*P* < 0.001) for H–O than O–H and fluctuated over time (*P* = 0.013).

## Discussion

The objective of this study was to measure gastric emptying in combination with metabolic and digestive responses in the plasma and cecum of horses when changing the feeding order of oats and hay. It was found that feeding order affected gastric emptying and that digestive and metabolic responses were clearly reflected in cecum pH than in plasma glucose and insulin. The methodological challenges and interpretation of the results are discussed below.

Digestive and metabolic responses are affected by several factors, e.g., diet composition (Julliand et al., 2001), digestibility of the diet, and retention time in the different segments of the gastrointestinal tract (Jensen et al., 2014, 2016). Several studies have investigated the digestive and metabolic responses of different diets fed to horses, e.g., fluctuations in plasma glucose and insulin as a measure of starch digestion in the small intestine (Vervuert et al., 2003) or changes in hindgut pH and SCFAs as a measure of fermentation of nutrients reaching the hindgut (Julliand et al., 2001; Thorringer et al., 2020). However, limited information exists on the effect of gastric emptying on the digestive and metabolic responses in horses when measured simultaneously.

The ^13^C breath test is a noninvasive technique for quantifying gastric emptying in horses and may be used as an alternative to, e.g., radioscintigraphy (Sutton et al., 2003). Gastric emptying, absorption from the small intestine and metabolism of a ^13^C-labeled substrate, results in ^13^C enrichment of exhaled CO_2_ that can be measured in collected breath samples as the ^13^C:^12^C ratio. In theory, the faster a response of ^13^C is measured in exhaled CO_2_ the faster the gastric emptying of the labeled substrate. Both acetic and octanoic acids have been used as labeled substrates for quantifying liquid- and solid-phase gastric emptying in horses, respectively (Wyse et al., 2001; Sasaki et al., 2005). In the present study, the ^13^C acetic acid was mixed with the oats and it is assumed that the ^13^C acetic acid followed the oats. However, when interpreting the results, it should be noted that the marker, in theory, follows the liquid phase connected to the oats, while the oats or the nutrients in oats (e.g., starch) are not labeled. Labeling the starch would have been optimal, but practically not possible. Despite this, it was possible to measure the effect of feeding order on gastric emptying.

The first hypothesis was that feeding oats before H would increase gastric emptying of oats compared with the opposite feeding order. The amount of time that passed before the maximal amount of ^13^C was excreted (*T*_max_) in exhaled air was increased when feeding H–O (2.2 h) in comparison to O–H (1.1 h), reflecting a possible delayed gastric emptying of oats when H was fed first. The horses were fed H at 2000 hours the evening before measurements. It could, therefore, be expected that oats fed before H in the morning would reach a relatively empty stomach after ingestion, in contrast to the opposite feeding order, where H is fed before the oats. In the latter, situation H might delay the gastric emptying of oats resulting in the later *T*_max_ for this feeding order. Saliva production in horses is estimated to be ~6 liters/kg of forage and 2 liters/kg of concentrate (Coenen et al., 2011). Given that the morning meals consisted of identical quantities of hay and oats, the total volume of saliva produced would be expected to be similar. Specifically, a meal comprising 1.5 kg of H and 0.5 kg of O would stimulate the production of ~10 liters of saliva. However, altering the order of feed intake could potentially influence the fluid dynamics within the horse’s digestive system, including the passage rate of ^13^C acetic acid along with the fluid phase through the stomach. These changes in fluid flow could, in turn, account for the observed variations in *T*_max_ and cecal pH as discussed below.

Vervuert et al. (2009) studied the effects of feeding state (feeding H or not, before and after a meal of CC (2 g starch/kg BW)) on the glucose and insulin responses. Here the highest peak glucose and insulin levels were measured when restricting H before, but not after feeding CC (like when feeding oats before hay in the present study). Furthermore, the lowest glucose and insulin peaks were found for feeding H before, but not after feeding CC (like the H–O feeding order in the present study). These differences could be due to differences in gastric emptying of the CC, as suggested in the present study.

The second hypothesis was that a rapid gastric emptying of O when feeding O before H would result in a faster metabolic response in plasma glucose and insulin. Hence, it was expected that plasma glucose and insulin would peak faster when feeding O before H compared with the opposite feeding order. However, there were no measured differences in plasma glucose or insulin responses in the present study. Insulin secretion is stimulated by a variety of different macronutrients like glucose, amino acids, and fatty acids, in addition to other stimulants like hormones, neural input, and humoral factors (Pørksen, 2002; Wilcox, 2005). Glucose and insulin responses are, therefore, highly individual. Even for horses of the same breed, size, and gender, where variations in measured glucose and insulin responses can be large, making it difficult to detect small differences, as shown in other studies investigating glucose and insulin responses (Vervuert et al., 2009; Thorringer et al., 2020). Moreover, glucose does not solely originate from the digestion of oat starch. The WSC in the H can also influence glucose and insulin response, making it challenging to isolate the specific impact of O. To enhance the measured effects of the feeding order of O on gastric emptying, it would be beneficial to include a greater number of animals in the study to mitigate the effects of individual differences. Supplying H with a reduced level of WSC could minimize its influence on the outcomes. The WSC content of the hay fed in the present study was 103 g/kg DM, and forages with a lower WSC content could have been used. Alternatively, providing a more substantial portion of starch-based feed, such as O, could amplify the metabolic reactions, improving the assessment of feeding order on gastric emptying and the resulting changes in plasma glucose and insulin levels.

Zeyner et al. (2004) studied the feeding sequence of concentrate (1.00 kg of oats/100 kg BW) and varying levels of forage (0.50, 0.67, 0.83, and 1.00 kg hay/100 kg BW) on different variables in feces (DM, pH, SCFA, ammonia, and buffering capacity), and concluded that feeding forage prior to concentrate appeared to have the potency to protect hindgut content from acidification. Philippeau et al. (2009) investigated the impact of feeding concentrate (ground barley, 1.75 g starch/kg BW) before or after forage on colonic pH in horses. They found that feeding concentrate before forage resulted in a lower colonic pH at 6- and 7-h post-feeding compared with when forage was fed before the concentrate. Jensen et al. (2012), on the other hand, did not find any differences in cecal pH when changing the feeding order of oats (1 and 2 g starch/kg BW) and forage. However, they did observe that minimum cecum pH was reached in a shorter time span when oats were fed before hay compared with the reverse feeding order (4 h 28 min vs. 6 h 28 min). Jensen et al. (2012) used continuous measurements of cecal pH and expressed the results in 10-min intervals, making it easier to detect variations in pH compared with, for example, hourly samples.

The third hypothesis was that a rapid gastric emptying of O, when feeding O before H, would result in a faster increase in cecal SCFA concentrations and a decrease in pH compared with the opposite feeding order. In the current study, horses were fed a relatively low amount of starch (0.4 g starch/kg BW/meal) when compared with general maximum recommendations (1 g starch/kg BW/meal) (Coenen et al., 2011). Despite this, results confirmed that feeding O before H may lead to a quicker reduction in cecal pH and, overall, a lower cecal pH than the reverse feeding order. These results were in accordance with those reported by Jensen et al. (2012). Over time, the proportion of acetate went down while propionate went up in the cecum, suggesting that starch was being fermented in the cecum (Julliand et al., 2001). These observations may indicate that starch reached the cecum faster when O were fed before H due to a more rapid gastric emptying and potentially faster passage rate through the small intestine. Sadet-Bourgeteau et al. (2016) found no clear effect of feeding order of concentrate and forage on cecal, colonic, and fecal pH, and SCFA concentrations (measured at 2 h intervals). The more frequent sampling intervals using continuous measurements of pH might detect smaller changes in the pH as shown by Jensen et al. (2012) and in the present study.

This study demonstrated that including different techniques can be very useful when evaluating the digestive and metabolic responses of different diets. Basic information regarding the effect of diet on gastric emptying, passage rate of digesta, and digestion is relevant for broadening our understanding of how different feedstuffs are digested and interact with one another. Furthermore, it is relevant for understanding the link between diet, health, and diseases. Gastric ulcers are related to diet and feeding management (Luthersson et al., 2009), and understanding normal gastric function is relevant for implementing appropriate feeding advice to horses with gastric ulcers. Research indicates that starch and sugars can undergo fermentation in the horse’s stomach, leading to the production of SCFA and potentially causing a reduction in pH levels, particularly in the nonglandular portion of the stomach (Métayer et al., 2004). This raises the question of whether such changes could contribute to the development of gastric ulcers in horses. Furthermore, the extent of this fermentation process may impact the energy value of feeds, which warrants further exploration. Therefore, additional research is needed not only to establish the degree to which this fermentation occurs but also to understand its implications for both gastric ulcer development and the determination of feed energy values. Lactic acidosis arises when dietary starch exceeds the digestive capacity of the small intestine, leading to fermentation in the cecum and colon. High starch fermentation and reduced cecal pH can detrimentally alter gut bacteria and increase lactic acid production, harming the cecal wall and elevating colic and laminitis risks in horses (Milinovich et al., 2010). Based on the results from the present study, demonstrating the effects of feeding order on gastric emptying and starch digestion, it may be advised that feeding order should be considered in practical feeding situations, especially if larger (>1 g starch/BW/meal) amounts of starch-rich concentrates are fed.

## Conclusion

The present study indicates that feeding order of H and O affected gastric emptying, and the digestive and metabolic responses were more clearly reflected in cecum pH than in plasma glucose and insulin. Feeding O before H decreased the *T*_max_ reflecting a faster gastric emptying than when feeding the opposite feeding order. Furthermore, feeding O before H resulted in a faster drop to minimum cecal pH than when feeding the opposite feeding order. It is recommended that feeding order should be considered in practical feeding situations, and starch-rich concentrate should not be fed before forage.

## Abbreviations

a, b, and c: regression constants
aADFom: acid detergent fiber assayed with a heat-stable amylase and expressed without residual ash;
AOAC: Association of Official Analytical Chemists
AP: ^13^C abundances (in atom %)
APE: ^13^C enrichments (in atom %)
AUC: area under the curve
BW: body weight
Cfat: crude fat
CP: crude protein
DM: dry matter
GEC: gastric emptying coefficient
aNDFom: neutral detergent fiber assayed with a heat-stable amylase and expressed without residual ash
N: nitrogen
PDR: percentage of ^13^C excretion in breath CO_2_ per hour
δ^13^C: relative delta value (in ‰) of ^13^C to ^12^C in breath CO_2_
SCFA: short-chain fatty acid
R: the international Pee Dee Belemnite standard (R = 0.0112372)
RF: the cumulative ^13^C excretion (in % of the ^13^C dose)
*t*: time
*t*_1/2_: time where half of the total cumulative recovery of ^13^C is excreted
*T*_max_: time where the maximal amount of ^13^C is excreted
